# The Membrane Proximal Domain of TRPV1 and TRPV2 Channels Mediates Protein–Protein Interactions and Lipid Binding In Vitro

**DOI:** 10.3390/ijms20030682

**Published:** 2019-02-05

**Authors:** Pau Doñate-Macián, Elena Álvarez-Marimon, Francesc Sepulcre, José Luis Vázquez-Ibar, Alex Perálvarez-Marín

**Affiliations:** 1 Biophysics Unit, Department of Biochemistry and Molecular Biology, School of Medicine, Universitat Autònoma de Barcelona, 08193 Cerdanyola del Vallés, Catalonia, Spain; pabdoama@gmail.com (P.D.-M.); elena.alvarez@uab.cat (E.A.-M.); 2 Departament d’Enginyeria Agroalimentària i Biotecnologia, Universitat Politècnica de Catalunya, 08860 Barcelona, Catalonia, Spain; francesc.sepulcre@upc.edu; 3 Institut de Biologie Intégrative de la Cellule, CEA-Saclay, 91191 Gif-sur-Yvette, France; jose-luis.vazquez-ibar@i2bc.paris-saclay.fr; 4 Institut des Sciences du Vivant Frédéric-JOLIOT, CEA-Saclay, 91191 Gif-sur-Yvette, France; 5 Institut de Neurociències, Universitat Autònoma de Barcelona, 08193 Cerdanyola del Vallés, Catalonia, Spain

**Keywords:** Transient Receptor Potential (TRP) channels, exocytosis, biophysics, protein–protein interactions, lipid-protein interactions

## Abstract

Constitutive or regulated membrane protein trafficking is a key cell biology process. Transient receptor potential channels are somatosensory proteins in charge of detecting several physical and chemical stimuli, thus requiring fine vesicular trafficking. The membrane proximal or pre-S1 domain (MPD) is a highly conserved domain in transient receptor potential channels from the vanilloid (TRPV) subfamily. MPD shows traits corresponding to protein-protein and lipid-protein interactions, and protein regulatory regions. We have expressed MPD of TRPV1 and TRPV2 as green fluorescente protein (GFP)-fusion proteins to perform an in vitro biochemical and biophysical characterization. Pull-down experiments indicate that MPD recognizes and binds Soluble N-ethylmaleimide-sensitive factor Attachment Protein Receptors (SNARE). Synchrotron radiation scattering experiments show that this domain does not self-oligomerize. MPD interacts with phosphatidic acid (PA), a metabolite of the phospholipase D (PLD) pathway, in a specific manner as shown by lipid strips and Trp fluorescence quenching experiments. We show for the first time, to the best of our knowledge, the binding to PA of an N-terminus domain in TRPV channels. The presence of a PA binding domain in TRPV channels argues for putative PLD regulation. Findings in this study open new perspectives to understand the regulated and constitutive trafficking of TRPV channels exerted by protein-protein and lipid-protein interactions.

## 1. Introduction

Membrane protein localization in the plasma membrane depends on trafficking mechanisms, such as exocytosis. Exocytosis is a regulated process that makes readily available a set of proteins in the plasma membrane of excitable and non-excitable cells. SNARE-dependent exocytosis in excitable cells involves proteins such as syntaxins (Stx), synaptotagmins (Syt), and snapins [1,2]. SNAREs coordinate an intricate protein-protein interaction network that facilitates vesicle docking and fusion with the plasma membrane. Soluble N-ethylmaleimide-sensitive factor Attachment Protein 25 (SNAP25) and plasma membrane anchored Stxs form a complex that allows vesicle docking by interacting with vesicle anchored Syts. Binding of Syts to Stxs-SNAP25 complex is a calcium-dependent process [3,4]. Syts present a C-terminal domain that mediates Stxs interaction upon calcium binding, acting as calcium sensors [3]. The Stxs C-terminal transmembrane domain is used to interact with voltage-gated calcium channels [5], which is a suitable molecular mechanism to ensure proximity between calcium gates and calcium-depedent vesicle fusion machinery. Snapin modulates exocytosis by enhancing the interaction of Syts with SNAP25 [2].

Transient receptor potential (TRP) channels are somatosensory proteins that require plasma membrane trafficking [6]. Some TRP channels act as mechanosensors, responding to physical and chemical stimuli affecting membrane mechanical properties [7]. Among these, the TRPV1–4 subgroup consists of four channels that respond to heat and osmolality changes, factors that affect membrane properties [8]. TRPV1–4 channels localize in the plasma membrane, being trafficked through regulated and constitutive pathways [6]. TRPV1 from the vanilloid subfamily [9] is trafficked via protein kinase C (PKC)—dependent exocytosis by interacting with synaptotagmin (Syt) I and IX, and snapin [10]. TRPV2, the closest homolog of TRPV1 also interacts with Syt-IX and snapin [11], and is also trafficked constitutively. For both SNAREs, the protein–protein interaction happens at the level of the cytosolic N-terminal region of TRPV1, most likely in the ankyrin repeat domain (ARD) and the neighboring pre-S1 membrane proximal domain (MPD) [10]. 

The ARD domain is a protein–protein interaction domain [12]; the MPD domain is, structurally, a link between the N- and C-terminus of the channel [13], and, functionally, is a temperature sensor in the TRPV1–4 subgroup [14]. The MPD is highly conserved among the TRPV1–4 subgroup. Through bioinformatics analysis, we have identified putative regulatory features such as phosphorylation sites, and lipid binding features in this MPD [15]. The current study aims to describe the biophysical determinants of the MPD domain, to understand whether this domain is capable of driving protein–protein and lipid–protein interactions.

## 2. Results

### 2.1. TRPVs and SNARE-Protein MPD-Mediated Interaction

**Figure 1:**
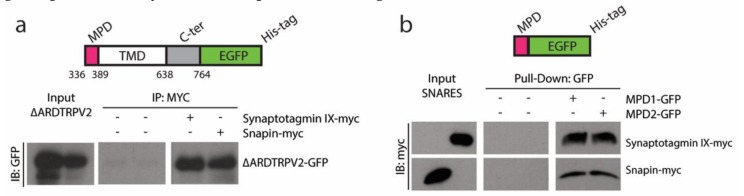
Mapping of the TRPV interaction with SNARE proteins. (**a**) Co-IP of human embryonic kidney 293 (HEK293) cells transfected with TRPV2 lacking N-terminal ARD domain GFP-tagged (ΔARD-TRPV2-GFP) and Snapin-25 or Synaptotagmin-IX c-myc-tagged. The lysates were immunoprecipitated with anti-c-myc. (**b**) In vitro pull-down assay using purified MPD region from TRPV1 or TRPV2 channels GFP-tagged to pull-down c-myc-tagged Snapin or Synaptotagmin-IX in lysates from HEK293 transiently transfected cells. MPDs-GFP were immobilized on GFP-Trap beads. IP: immunoprecipitation; IB: immunoblotting. Co-IP experiments have been carried out in the absence (−) and presence (+) of c-myc antibody to assess the specificity of the interaction.

The interaction between TRPV1–2 and SNARE proteins such as SytIX and Snapin has been previously described [10,11]. In Figure 1a, we show the interaction of a TRPV2 ARD-less truncation (ΔARD-TRPV2-GFP) with SytIX and Snapin, arguing for the existence of another domain in the N-terminus capable of driving the interaction rather than ARD. We use a pull-down approach to assess whether MPD is enough to drive the interaction between TRPV1–2 and SytIX and Snapin (Figure 1b), by using the TRPV1 and TRPV2 MPD (further called MPD1 and MPD2, respectively). MPD1 and MPD2 have been expressed recombinantly as GFP-fusion proteins following a two-step purification process (Figure S1). The pull-down experiment using GFP-trap beads indicates that the MPD is enough to pull-down SytIX and Snapin in vitro (Figure 1b).

### 2.2. MPD-Mediated Lipid Binding

**Figure 2:**
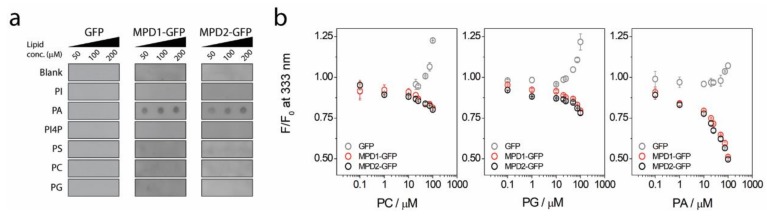
Interaction of MPD domain from TRPV1 and TRPV2 with phosphatidic acid (PA). (**a**) Blank (only buffer), PI, PA, PI4P, PS, PC, or PG lipids were immobilized on nitrocellulose membranes at concentrations (conc.) of 50, 100, or 200 μM. Purified recombinant GFP or MPD-GFP from TRPV1 or TRPV2 channels were incubated on the lipid-containing nitrocellulose membranes and detected using an anti-GFP antibody. (**b**) Tryptophan quenching experiments. GFP tryptophan fluorescence (emission at 333 nm) of free GFP or MPDs from TRPV1 or TRPV2 was monitored in the presence of increasing concentrations of the different lipid polar head-groups (Phosphatidylcholine, POPC, 1,2-Dipalmitoyl-sn-glycero-3-phosphate (DPPA), and Phosphatidylglycerol (POPG)) liposomes.

Taking advantage of the recombinant expression of the MPD1 and MPD2 GFP fusion proteins, we decided to test the lipid binding to MPD hypothesis from our previous study [15]. MPD1 and MPD2 were incubated with several lipids immobilized on a nitrocellulose membrane, such as phosphatidylinositol (PI), phosphatidylinositol 4-phosphate (PI4P), phosphatidic acid (PA), phosphatidylcholine (PC), phosphatidylserine (PS), and phosphatidylglycerol (PG). From all the headgroups tested, both MPD GFP-fusion proteins bind to PA in a concentration-dependent manner (Figure 2a). The GFP control did not bind to PA or any other lipid (Figure 2a). To confirm this result, we took advantage of the Trp residues present in the MPD but not in GFP. We monitored Trp fluorescence while incubating the MPD proteins with increasing concentrations of PC, PA, and PG liposomes (Figure 2b). Independently of the lipid headgroup, when GFP is incubated with increasing concentrations of liposomes, we observed a light scattering effect because of liposome aggregation (Figure 2b). In the presence of the MPD incubated with PC or PG liposomes, a slight Trp quenching is observed (circa (ca.) 25%). When MPD is incubated with PA liposomes, Trp quenching is about 60% at the highest liposome concentration, indicating a binding of PA liposomes to both MPD1 and MPD2 (Figure 2b).

### 2.3. MPD Oligomerization State

**Figure 3:**
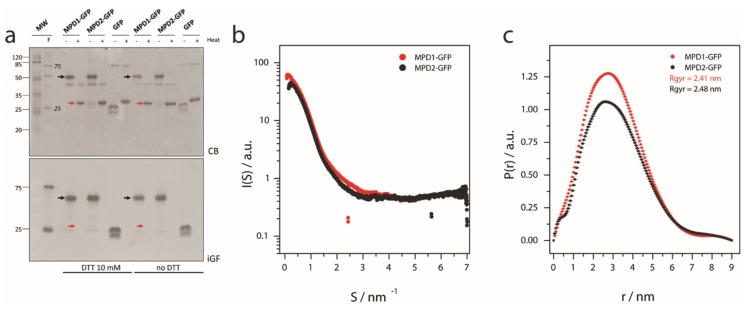
Oligomerization state of MPD domain for TRPV1 and TRPV2. (**a**) SDS-PAGE of boiled and not boiled MPD-GFP samples (heat +/−, respectively) and in the presence/absence of reducing agents observed by Coomassie blue (CB, top) and in-gel fluorescence (iGF, bottom; F stands for fluorescent molecular weight (MW) marker). Black and read arrows indicate the oligomeric and monomeric bands, respectively. (**b**) SAXS scattering plot for the MPD-GFP constructs for TRPV1 and TRPV2. (**c**) Probability distribution function for the MPD-GFP constructs for TRPV1 and TRPV2.

During the MPD-GFP purification, an apparent molecular weight (MW) of ca. 50 kDa was observed for the MPD-GFP constructs. The expected MW for these constructs was ca. 35 kDa, i.e., 27 kDA GFP( plus 8 kDa for the MPD. Dimerization of GFP proteins is described in the literature [16], so we compared GFP alone against our MPD-GFP constructs in an sodium dodecyl sulfate polyacrylamide gel electrophoresis (SDS-PAGE) (Figure 3a). In non-heated samples, Coomassie blue and in-gel fluorescence show a GFP monomeric band at ca. 27 kDa, and MPD-GFP constructs at ca. 50 kDa. Reducing agents such as dithiothreitol (DTT) could not disassemble the MPD-GFP high-molecular weight species (Figure 3a). Sample boiling for 5–10 min in Laemmli buffer dissolved these high-molecular weight species (as observed in Coomassie blue staining) but also prevented the observation of GFP-fluorescence (Figure 3a). Figure 3a indicates the possibility that the MPD is driving oligomerization, which could be an artifact promoted by high protein concentration. Synchrotron small-angle X-ray scattering (SAXS) data shows that no MPD-GFP construct is aggregated in solution (Figure 3b), and the probability function indicates that the radius of gyration (Rgyr) for both constructs is centered at ca. 2.5 nm (Figure 3c), as is the expected Rgyr for a monomeric MPD-GFP protein.

## 3. Discussion

**Figure 4:**
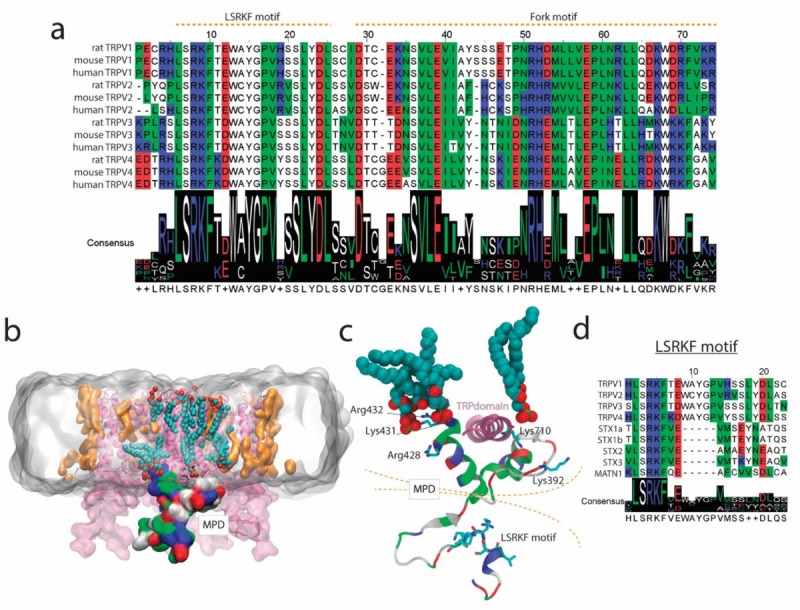
(**a**) The MPD domain is highly conserved within the TRPV1–4 subgroup. An orange dotted line delimits the LSRKF and Fork motifs. (**b**) Illustrative tridimensional depiction of TRPV1 (protein data bank (PDB) code 5IRZ, in pink surface representation) embedded in a DPPC/DPPA bilayer (grey surface) resulting from a membrane molecular dynamics (MD) equilibration trajectory (1 nanosecond). The MPD domain for Chain A is depicted as a colored surface. DPPA molecules within 5 Å of the protein are depicted in orange, except for those DPPA molecules within 5 Å of Chain A for TRPV1, which are depicted as Van der Wals (VdW) spheres colored by element (C in cyan; P in golden; O in red). (**c**) Representation of the MPD of TRPV1 highlighting the residues interacting with DPPA molecules. (**d**) Multiple sequence alignment for human sequences containing the LSRKF motif (STX, syntaxin; matrillin 1 or cartilage matrix protein (MATN1)). The color code for MSA and structure residue representation is the following: red, negatively charged; blue, positively charged; white, polar non-charged; green, non-polar.

Here we present our strategy to produce a recombinant fusion protein (Figure S1) containing the conserved MPD domain for the TRPV1–4 subgroup (Figure 4a). The biophysical characterization of the MPD domain in vitro indicates the potential of this domain to drive certain function at the cellular level. Initial bioinformatics predictions [15], such as protein-protein and lipid-protein interactions have been confirmed in vitro using biochemical and biophysical methods (Figure 1; Figure 2). Our results indicate that the MPD is not an oligomerization domain per se (Figure 3), but its role in the oligomerization of the channel in the context of the whole sequence cannot be ruled out, as discussed below.

TRPV channels are trafficked via constitutive and SNARE-dependent regulated exocytosis [6]. TRPV1 and TRPV2 have been shown to interact with SNARE binding vesicular proteins, such as SytIX and Snapin [10,11]. Our data indicate that the conserved MPD is involved in this interaction. Derived from our data and taking into account the sequence conservation within the MPD and the three-dimensional structure of TRPV channels, there is a region within the MPD, which could be a putative SNARE-binding protein motif (Figure 4a). The MPD consists of two structural regions, the Leu-Ser-Arg-Lys-Phe (LSRKF) motif (in the N-terminal region of the MPD) and the more hydrophobic bilayer proximal fork-domain interacting with the TRP domain (Figure 4a–c). The LSRKF motif contains the LSRKF fingerprint, which can be found in a few human proteins (Figure 4d), among those, TRPV1–4 and syntaxins (STX) 1a, 1b, 2, and 3. STX1a and STX1b are present in excitatory cells, whereas STX2 and STX3 are ubiquitously expressed [17], but in any case they bind to SNARE proteins such as Syt and Snapin. The LSRKF motif is present in the exocytosis-specific N-terminal domain of the mentioned STXs [18]. Further biochemical studies comparing STX and MPD peptides could explain the role of the LSRKF motif and protein–protein interactions, key to understanding the SNARE-dependent exocytosis/trafficking of TRPV channels.

It is known that STX1a interacts with high affinity with the phospholipase D signaling metabolite PA via a polybasic domain [19]. The hydrophobic fork-domain within the MPD (Figure 4a) contains a polybasic region putatively capable of lipid binding [15]. Research on TRPVs has shown that the C-terminus region of TRPV channels binds PA [20]. TRPV1 Lys710 in the TRP-domain has been shown to interact with PA, modulating its activity. Our study, although in vitro, is the first to show interaction of an N-terminus region of TRPV1 and TRPV2 with PA (Figure 2). In our study, the interaction of the MPD with PA is higher than with PC or PG, especially from the Trp quenching experiments. Although both PA and PG have negatively charged polar headgroups, the stronger interaction with PA may come from the smaller headgroup for PA, but also from the higher reactivity of PA as a function of pH [21]. In Figure 4c, we show a model for TRPV1 embedded in a dipalmitoylphosphatidylcholine (DPPC)/DPPA lipid bilayer. This model shows that, among the positively charged residues in the fork domain, Arg428, Lys431, Arg432, and Lys710 (in the C-terminus) in TRPV1 are within interaction distance with DPPA molecules (Figure 4c).

As most channels [22], TRPV1–4 channel function is modulated by lipid composition, especially by Phosphatidylinositol 4,5-bisphosphate (PIP2) through phospholipase C [23,24]. PA, as a cone shape lipid, exerts dramatic effects on membrane curvature and membrane fusion properties [25,26], key parameters to drive exocytosis. PA binding domains, such as the MPD in TRPV1–4 channels may be responsible for stabilizing the channel on closed conformation in PA-rich membranes, as happens with certain potassium channels [27]. Taking into account that TRPV1–4 cation channels are heat and mechanosensors [7], these proteins require an inactivation mechanism preventing cation flux through the channel during trafficking, which may lead to cation-toxicity (e.g., intracellular Ca^2+^ load). Thus, the strong specific binding of the TRPV1 and TRPV2 MPD in vitro (Figure 2) may indicate that MPD–lipid interaction is critical for the proper trafficking of the protein. In fact, the MPD domain is critical for the folding, assembly, and trafficking of oligomeric TRPV4 channels [28] via the interaction with the TRP domain (for clarity see Figure 4b,c). Our results indicate that the MPD domain without the TRP domain is not capable of driving self-oligomerization as shown by our SAXS data indicating monomeric species (Figure 3b,c). Further, in vitro characterization of MPD-TRP domain oligomerization could be relevant to determine the inter-domain interaction potential within the N- and C-terminus in TRP channels.

As a summary, we provide an in vitro biophysical characterization of the MPD for TRPV1 and TRPV2, a conserved domain among TRPV1–4 channels, revealing protein-protein and lipid-protein interaction features, which could be translated into the understanding of the molecular mechanism of these somatosensory channels in their physiopathological modulation by lipids [29,30], protein binding [11], and trafficking [6].

## 4. Materials and Methods

### 4.1. DNA Plasmids and Cloning

TRPV2 cDNA were cloned into a pcDNA3 vector in frame with an enhanced-GFP (eGFP) and 8XHis-tags at the C-terminus. The N-terminus truncation for TRPV2, ΔARD-TRPV2 (Δ1-336 rat TRPV2), was cloned by high-fidelity PCR amplification followed by ligation into the same pcDNA3 vector within NdeI and NotI sites. The MPD for TRPV1 and TRPV2 (MPD) sequences were purchased from GenScript (New Jersey, NY, USA) and cloned into pTTQ18-superfolder-GFP (sfGFP) plasmid within EcoRI-PstI sites.

### 4.2. Recombinant Protein Expression and Purification

MPD1 or MPD2 were overexpressed as fusion proteins containing an Human Rhinovirus 3C Protease (HRV3C) cleavage site followed by the sfGFP and a decahistidine tag (10× His) in the C-terminal. Proteins were expressed in *E. coli* BL21 cells in Luria Bertani (LB) media supplemented with ampicillin, and induced with 1 mM isopropyl β- D -1-thiogalactopyranoside (IPTG) at OD_600_ ≈ 0.6, overnight at 37 °C. Cells were collected by centrifugation (4000× *g* for 30 min), resuspended in 20 mM Tris·HCl (pH 8), 150 mM NaCl, 5% glycerol, pelleted again at 4000× *g* for 30 min, and stored at −80 °C. For lysis, cell pellets were resuspended in 20 mM Tris·HCl (pH 8), 150 mM NaCl, 5% glicerol, 2 mg/mL lysozyme, supplemented with protease inhibitors (0.5 µg/mL pepstatin, 1 mM 4-(2-aminoethyl)benzenesulfonyl fluoride hydrochloride (AEBSF), 5 mM benzamidine, and 1 Complete EDTA-free tablet (Roche, Germany) for each 50 mL), and stirred on ice for 20 min at 4 °C. Cell suspension was sonicated for 5 cycles of 30 s pulse followed by 30 s pause. The resulting lysate was centrifuged for 30 min at 24,000× *g* and the supernatant was collected and filtered through a 45 µm filter (Millipore, Germany). For purification, Talon (GE Healthcare, Germany) beads were equilibrated with 20 mM Tris·HCl (pH 8), 150 mM NaCl, 5% glicerol, and 5 mM imidazole and incubated with the filtrated supernatant for 1 h at 4 °C in stirring. Beads were washed with 20 column-volumes of 20 mM Tris·HCl (pH 8), 150 mM NaCl, 5% glicerol, and 20 mM imidazole, and proteins were eluted with 6 column-volumes of 20 mM Tris·HCl (pH 8), 150 mM NaCl, 5% glicerol, and 250 mM imidazole. The eluted protein was concentrated using a Centricon filter (3 kDa MW, Sartorius, Germany) to a final volume of 500 µL.

### 4.3. Cell Cultures and Transfection

HEK293 cells were cultured in Dulbecco’s modified Eagle’s medium (DMEM, Gibco, Spain) supplemented with 10% fetal bovine serum (FBS), 100 units/mL penicillin, and 100 μg/mL streptomycin. Transfection was performed using polyethyleneimine (PEI, Polysciences, 23966, Germany). HEK293 cells overexpressing the transfected constructs were lysed 48 h after transfection, and membrane proteins were solubilized for 30 min at 4 °C in lysis buffer (50 mM Tris-HCL pH 7.4, 150 mM NaCl, 2 mM EDTA, 1% Triton, 5% glycerol, 1 mM benzamidine, and EDTA-free protease inhibition cocktail, ROCHE 11873580001, Germany). Cell extracts were centrifuged at 14000× *g* at 4 °C for 10 min to remove aggregates. 

### 4.4. Immunoblotting

Lysates and immunoprecipitates were loaded into SDS-page gels and run at 100 mV for 90 min. Gels were transferred to nitrocellulose membranes into a semi-dry cast at 100 mA for 1 h. Membranes were blocked in blocking buffer (5% non-fat-dry milk TTBS 1×) ON at 4 °C. Primary antibodies were incubated in blocking buffer for 1 h at room temperature. Primary antibodies were diluted as follows: anti-MYC tag (551101, Pharmingen, Germany) 1:1000, anti-GFP tag (GFP-G1, DSHB, Iowa, IA, USA) 1:1000. Secondary antibodies were incubated in blocking buffer for 1 h at RT. Anti mouse (sc-2031, SantaCruz, Dallas, TX, USA) and anti rabbit (sc-2030, SantaCruz, Dallas, TX, USA) were used at 1:2000. Membranes were developed with Luminata crescendo reagent (WBLUR0100, Millipore, Germany). 

### 4.5. Co-Immunoprecipitation

Soluble fractions from cell lysates were used as input for co-immunoprecipitations. Cell extracts at 1 µg/ul (500 µg total protein) were incubated overnight at 4 °C with anti-MYC antibody (551101, Pharmingen, Germany). Immuno-complexes were then incubated with 50 uL of sepharose beads (17-0618-01, GE Healthcare, Germany) for 2 h at 4 °C. After incubation, the complexes were washed with lysis buffer 3 times. Immunoprecipitated complexes were then denatured with SDS-PAGE sample buffer (90 °C for 5 min), separated by SDS-PAGE and analyzed by western blotting. 

### 4.6. Pull Down

MPD from TRPV1 and TRPV2 were purified from *E. coli* as described above. Purified MPDs were added to Hek293 cell lysates from Snapinand Synaptotagmin-IX MYC-tagged transfected cells. Cell extracts at 1 ug/uL (500ug total protein) were incubated 2 h at 4 °C and then incubated with 50 uL of GFP-trap A beads (gta-10, Chromotek, Germany) for 2 h at 4 °C. After incubation, the beads were washed with lysis buffer 3 times, denatured with SDS-PAGE sample buffer (90 °C for 5 min), separated by SDS-PAGE, and further analyzed by immunoblot.

### 4.7. Lipid Strips

Lipids were resuspended in a 1:2:1 parts Chloroform-Methanol-Water buffer. Solubilized lipids were diluted in 1:2:1 parts of a Chloroform-Methanol-Tris·HCl 50 mM buffer and spotted into a nitrocellulose membrane at the desired final concentrations of 0, 50, 100, or 200 μM. Membranes were dried at RT for 1 h and then stored at 4 °C ready to use. Membranes were blocked for 1 h at RT with blocking buffer (2% bovine serum albumin (BSA) PBS 1×) and incubated with purified protein, free GFP, MPD1-GFP, or MPD2-GFP, at 9.5 μg/mL in a blocking buffer for 1 h at RT. After incubation membranes were washed 3 times in Tween20- tris-buffered saline TTBS 1× and further analyzed by immunoblot against a GFP tag. 

### 4.8. Tryptophan Quenching

For the liposomes generation, we departed from a chloroform solution of each phospholipid. The required volume was deposited on a ball rotavapor (Rotavapor R-3000, Büchi, Switzerland) to obtain a final concentration of 1.5 mM lipid in the final suspension. The solution was dried in a rotavapor to obtain a film of phospholipid, which was further resuspended in 2 mL/ball of 50 mM Tris-HCl (pH 7.4), 150 mM NaCl, 10% glycerol, and EDTA-free protease inhibitors. Agitation in a rotavapor was performed in order to hydrate phospholipids to form a suspension containing large multilamellar liposomes. Tryptophan endogenous fluorescence, excitation at 280 nm and emission at 332 nm, was measured using a PTI Quantamaster fluorimeter (PTI, Canada). Proteins in 50 mM Tris-HCl (pH 7.4), 150 mM NaCl, and 10% glycerol were incubated in a quartz cuvette and lipids reconstituted in the protein buffer were added at increasing concentrations. 

### 4.9. SAXS Data

SAXS data were collected at the Non-Crystalline Diffraction beamline at the ALBA-CELLS synchrotron light source, (Cerdanyola del Vallés, Barcelona, Spain). For each protein (20 mg/mL), 60 frames of a 1 s exposure time were collected. The X-ray beam energy was at 12.4 keV, and the sample to detector distance was 6.25 m. Background was checked before and after each frameset to monitor radiation damage.

Initial data reduction was carried out using SAXSIT [31]. Images were circularly integrated to yield an averaged and reduced linear I(S) versus S plots. All the scattering measurements were checked before the averaging and subtraction of the buffer solution. Data were corrected for sample transmission, background, and detector sensitivity and were normalized to the scattering cross-section per unit sample volume I(S). PRIMUS and GNOM software [32,33] was applied to process the experimental data and further to evaluate the P(r). Ab initio modeling was done using DAMMIN software [34]. PRIMUS, GNOM and DAMMIN are available at https://www.embl-hamburg.de/biosaxs/. 

### 4.10. MSA and Tridimensional Representations

LSRKF motif containing sequences have been identified using BLASTP [35] against human sequences. MSA was performed with MAFFT [36] and depicted using JalView [37]. Protein-membrane bilayer system was prepared in CHARMM-GUI [38]. Tridimensional structure representations have been done using VMD [39].

## Supplementary Materials

Supplementary materials can be found at http://www.mdpi.com/1422-0067/20/3/682/s1.

## Abbreviations

MPD	Membrane proximal domain
TRP	Transient receptor potential
TRPV	Transient receptor potential vanilloid
SNARE GFP Stx Syt PLD PA PI PI4P PC PS PG	SNAP (Soluble N-ethylmaleimide-sensitive factor Attachment Protein) REceptor Green fluorescent protein Syntaxins Synaptotagmin Phospholipase D Phosphatidic acid Phosphatidylinositol Phosphatidylinositol 4-phosphate Phosphatidylcholine Phosphatidylserine Phosphatidylglycerol
