# Supplementary material for: The Membrane Proximal Domain of TRPV1 and TRPV2 Channels Mediates Protein–Protein Interactions and Lipid Binding In Vitro
Source: Int J Mol Sci. 2019 Feb 5;20(3):682. doi: 10.3390/ijms20030682 (PMC6387362; doi:10.3390/ijms20030682)
Supplement: Supplementary file 1 [file ijms-20-00682-s001.pdf]

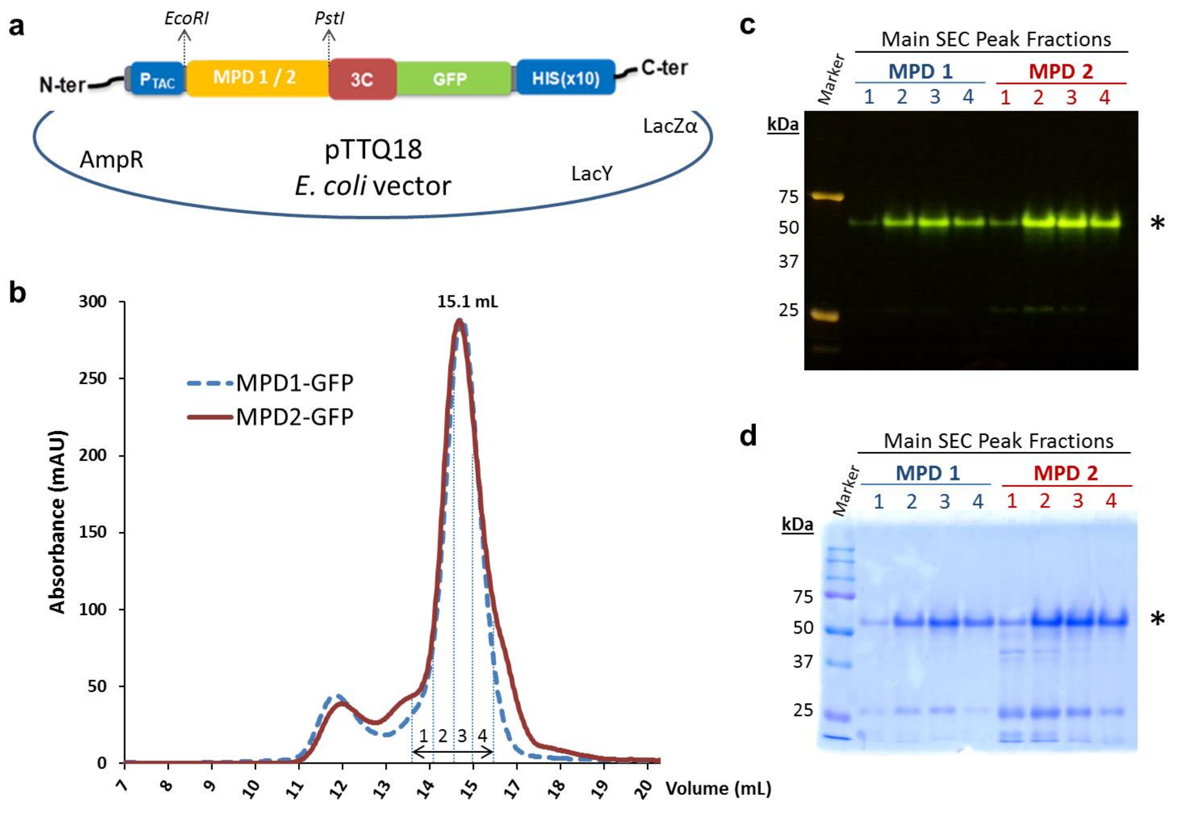

Figure S1. MPD-GFP expression and purification strategy. A) MPD1 or MPD2 expression vector scheme. MPD1 or MPD2 genes were cloned *in frame* using EcoRI and PstI restriction enzymes into the *E.coli* pTTQ18-C3-GFP-His vector. The resulting fusion protein would contain a C3 PreScission cleavage site followed by the superfolder Green Fluorescent Protein (sf-GFP) and a 10x Histidine C-terminal tag. **B) Size Exclusion Chromatography (SEC) of purified MPD1/2-GFP-His proteins.** The purified MPD1/2-GFP-Hisproteins by Ni^{++}-NTA affinity chromatography column were run into a Superdex 200 10/300 SEC column and both proteins show a main elution peak centered at a 15.1 mL volume (blue dashed line corresponds to MPD1-GFP-His and red line to MPD2-GFP). This result is compatible with a ca. 50 kDa molecular size protein that would correspond to a monomer of the fusion protein. **C and D)** The SEC fractions 1 to 4 were collected and run into an SDS-PAGE electrophoresis using a 10 % polyacrylamide gel. **C) In Gel Fluorescence** of the fractions 1 to 4 corresponding to the main peak of the SEC of both purified proteins MPD1 or MPD2 fusion proteins. The "*****" shows the fluorescent protein that fits to the molecular weight of a dimer of MPD1/2-GFP fusion proteins. Non-boiled samples run as a dimer and boiled ones run as a monomer in SDS-PAGE electrophoresis (see figure 3). **D) Comassie staining** of the same SDS-PAGE gel showed the purity of the protein sample (*) and small amounts of free GFP and some impurities that could be removed with a second purification step.
